# Drug Safety Assessment Based on Target Affinity, Drug Exposure and Plasma Protein Binding: Drug-Induced Cardiotoxicity from a Translational Pharmacology Perspective

**DOI:** 10.3390/ijms27104563

**Published:** 2026-05-19

**Authors:** Simona Catozzi, Fianne Sips, Niccolò Totis, Marc-Antonio Bisotti, Sofia Stathopoulos, Mario Torchia, Luca Emili, Vincenzo Carbone, Candice Baker, J. Matthew Mahoney, Daniel Röshammar

**Affiliations:** 1 InSilicoTrials Technologies S.p.A., 34123 Trieste, Italy; simona.catozzi@insilicotrials.com (S.C.); niccolo.totis@insilicotrials.com (N.T.); marc-antonio.bisotti@insilicotrials.com (M.-A.B.); sofia.stathopoulos@insilicotrials.com (S.S.); mario.torchia@insilicotrials.com (M.T.); luca.emili@insilicotrials.com (L.E.); vincenzo.carbone@insilicotrials.com (V.C.); 2 InSilicoTrials Technologies B.V., 1017 BR Amsterdam, The Netherlands; fianne.sips@insilicotrials.com; 3 The Jackson Laboratory, Bar Harbor, ME 04609, USA; candice.baker@jax.org (C.B.); matt.mahoney@jax.org (J.M.M.)

**Keywords:** DICTrank, cardiovascular safety, target affinity, drug exposure, plasma protein binding, drug discovery and development

## Abstract

Cardiac safety assessment is an integral part of drug discovery and development. Drug candidates that adversely affect cardiac or hemodynamic function should be discontinued early unless a favorable benefit-risk ratio for patients can be justified. In this hypothesis-generating work, we aimed to develop a conceptual framework for informing early safety risk assessment based on in vitro drug affinities to pharmacological targets. For illustration, we used the drug-induced cardiotoxicity rank (DICTrank) data comprising 1318 drugs with cardiac safety concerns according to FDA labeling. The data was enriched with information on affinity to the most plausible mechanistic targets, clinical drug exposure, and human plasma protein binding. We descriptively identified 18 target classes potentially associated with elevated cardiovascular risk: potassium channels (accounting alone for 20% of the ‘most concern’ safety group); adrenergic, dopamine, serotonin, androgen, sex hormone, and opioid receptors; cyclooxygenase; sodium and calcium channels; muscarinic and glucocorticoid receptors; phosphodiesterase; topoisomerase; angiotensin-converting enzyme; angiotensin II type 1 receptor; monoamine transporters, and acetylcholinesterase. Overall, 80% of the ‘most concern’ drugs compared with only 12% of the ‘no concern’ drugs were associated with these targets in this exploratory descriptive analysis. Concentration–response analyses revealed differences in target potency and free drug exposure that appeared associated with variability in the severity of cardiotoxicity among drugs acting on the same target. This framework demonstrates how in vitro data can be used to benchmark new compounds early in development, enabling the timely discontinuation of candidates associated with substantial risk.

## 1. Introduction

Cardiac safety assessment is an integral part of drug discovery and development. Drug candidates with the potential to adversely affect cardiac or hemodynamic function should be discontinued early unless a favorable benefit-risk ratio for patients can be justified. Historically, cardiac toxicity has been one of the leading causes of safety-related attrition in both preclinical and clinical research [1].

The drug-induced cardiotoxicity (DICT) rank (DICTrank) database comprises 1318 drugs with and without evidence of cardiac safety concerns based on FDA labeling. Drugs are classified into four concern categories (most, less, no, ambiguous) and further stratified by severity (mild, moderate, severe). The database spans a wide range of therapeutic classes and represents one of the most comprehensive curated resources of cardiotoxicity, providing a valuable foundation for the development of novel approach methodologies [2]. Numerous studies have investigated factors contributing to the toxicity among the DICTrank drugs. Interestingly, Mukerjee et al. identified various molecular and physicochemical properties related to drug absorption, distribution, metabolism, excretion and toxicity (ADMET) that were predictive of adverse events. These factors included substrate status for cytochrome P450 (CYP) 2D6, inhibition of CYP2D6 and CYP1A, concomitant drug-induced liver injury, aqueous solubility and presence of aromatic moieties in the molecular structure [3]. While some of these features may indirectly influence drug exposure and target engagement, the causal relationships between these properties and specific adverse events remain insufficiently understood.

In contrast, Seal et al. incorporated more mechanistically orientated predictors derived from biological and chemical data, including molecular structural alerts and peak plasma drug concentration [4]. However, a detailed pharmacological interpretation linking these predictors to the underlying mechanisms of adverse events was not provided.

We note that machine learning-based predictions can likely be substantially strengthened by integrating insights about underlying pharmacological pathways. Characterizing the relationship between drug exposure and biological response may help explain why some drugs elicit more adverse reactions than others. While machine learning methods have proven valuable in retrospective analyses, their ability to reliably predict outcomes in novel scenarios, beyond the conditions represented in the training data, is more limited. To address this challenge, we advocate for more systematic, mechanistically informed approaches. Exploration of large sets of molecular properties and other covariates should be accompanied by a clear rationale, explaining why these features are plausible predictors of toxicity.

A key initial step in assessing drug safety (and efficacy) is understanding the mechanism(s) of action. A fundamental question is whether a molecule has affinity for targets known to be associated with cardiotoxicity. Drug responses (both therapeutic and adverse) most often arise from interactions with one or more intended on-targets and unintended off-targets, such as receptors, ion channels, enzymes, or transporters. Binding of a drug to its target may result in either activation or inhibition of the target’s function. Because the physiological systems governing cardiovascular function are well characterized, the downstream pharmacological consequences of modulating these targets can often be anticipated. Importantly, in vitro measurements of target affinity can be generated early in drug development, enabling assessment of cardiovascular risk without the immediate need for animal experiments.

A second key factor in toxicity prediction is dosing, encompassing both the amount and frequency of drug administration. As stated by Paracelsus: “What is there that is not a poison? All things are poison, and nothing is without poison. Only the dose determines that a thing is not a poison” [5]. Accordingly, drug exposure relative to target potency determines whether therapeutic efficacy can be achieved without exceeding acceptable safety limits. Characterizing target engagement over time, along with the exposure–response relationship, is therefore critical for study design and dose selection. In addition, the potential impact of between-subject variability should also be explicitly considered when evaluating safety margins and defining therapeutic windows.

Third, only the unbound (free) fraction of drug is generally considered pharmacologically active, capable of binding to its target and eliciting a biological response [6]. Although total drug concentrations are often measured in clinical settings for practical reasons, differences in unbound fraction across species, in vitro systems, and humans must be taken into account when translating target affinities and exposure–response relationships. It is therefore essential to assess human plasma protein binding early in drug development, as this enables more accurate interpretation of exposure–response and supports reliable translational scaling.

Adverse cardiovascular drug reactions can often be anticipated based on key pharmacological properties such as target affinity, drug exposure, and plasma protein binding. These predictors align with the ‘right target/efficacy’, ‘right tissue/concentration’ and ‘right safety’ pillars of a five-dimensional framework for drug development that was previously presented based on learnings from multiple programs and portfolios [1]. By increasing the use of in vitro data, animal studies can be better informed and, in some cases, further reduced when addressing these type of questions.

A similar concept has previously been proposed for the assessment of pro-arrhythmic drug safety risks [7]. In that work, the authors demonstrated how the clinical risk of QT interval prolongation can be predicted using in vitro data on ion channel inhibition. Moreover, subsequent studies have shown that in silico drug trials can outperform animal studies in predicting pro-arrhythmic risk [8]. In the present work, we propose extending this framework to encompass a broader spectrum of adverse cardiovascular drug reactions beyond QT interval prolongation. We illustrate this concept by systematically exploring the underlying pharmacological mechanisms of drugs included in the DICTrank database. Leveraging recent advances in artificial intelligence (AI), we employed a biomedical agent to summarize, curate, and integrate complementary pharmacological information from publicly available sources.

The objective of the present hypothesis-generating work was to develop a conceptual framework for safety risk assessment based on in vitro drug affinities to pharmacological risk targets in conjunction with corresponding free drug exposure levels. This framework is intended to enable early prediction of clinically relevant dosing regimens and benefit-risk profiles, with the potential to prevent failed animal and clinical studies. Guided by these principles, we performed a descriptive analysis of the drugs included in the DICTrank database to explore potential associations between cardiovascular toxicities and affinities for common cardiovascular drug targets. The specific aim of the analysis was to identify key potential cardiovascular risk targets (such as specific receptors, ion channels, enzymes, and transporters) that may be suitable for early-stage in vitro-based safety screening and risk assessment of new drug candidates.

## 2. Results

In total, 1267 unique generic drug names from the DICTrank database were included in the analysis. In the original dataset with 1318 entries, several drugs appeared multiple times under different trade names or salt forms and were therefore consolidated. The drugs were distributed across the DICT concern categories as follows: most concern (n = 327), less concern (n = 507), ambiguous concern (n = 105) and no concern (n = 328). Each drug was descriptively mapped to a pharmacological target (or absence thereof) that could plausibly explain the cardiovascular event reported in DICTrank, based on a structured review of the literature. Data on plasma protein binding, target affinity (defined as the concentration producing half maximal engagement [EC_50_ or IC_50_]), and maximum clinical plasma drug concentrations (C_max_) were retrieved for 81.1%, 73.8 and 53.5% of the drugs, respectively. Complete data across all variables were available for 581 drugs.

### 2.1. Overview of Pharmacological Targets Identified for the DICTrank Drugs

**Figure 1:**
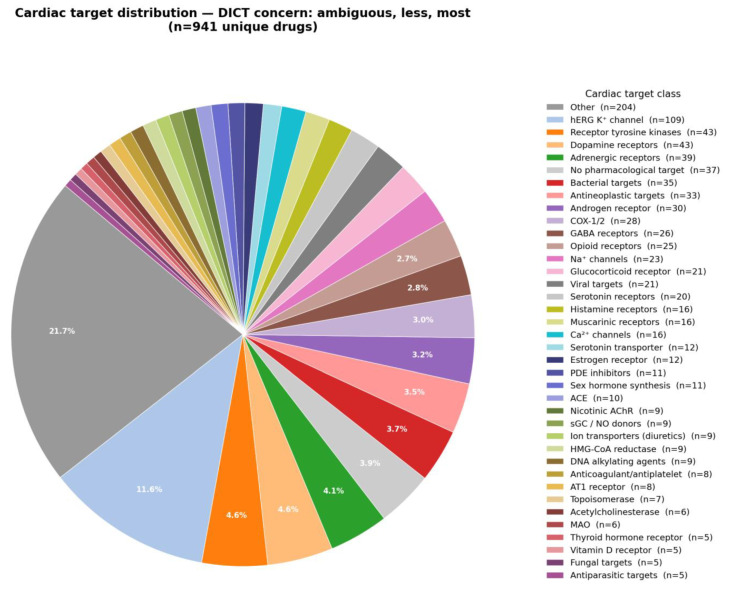
Targets associated with the various DICTrank drugs presenting with any concern (most, less or ambiguous).

**Figure 2:**
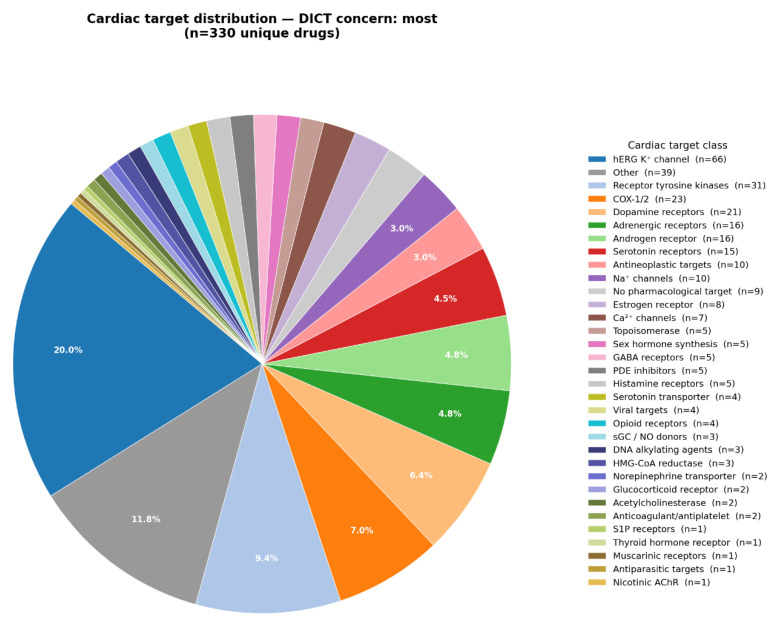
Targets associated with the various DICTrank drugs presenting with most concern.

The drug targets associated with any level of DICT concern are summarized in Figure 1. Targets that occurred less frequently were grouped together into an ‘Other’ category. However, many of these may still have plausible mechanistic links to adverse cardiovascular events. Notable examples include adenosine receptor agonists and antagonists (e.g., adenosine, regadenoson, caffeine), calcineurin inhibitors (tacrolimus, voclosporin), calcium-sensing receptor agonists (cinacalcet, etelcalcetide), inhibition of the hyperpolarization-activated cyclic nucleotide-gated (HCN) channel pacemaker current (ivabradine), and the classical Na^+^/K^+^-ATPase inhibitor digoxin. Drug targets associated with the most DICT concern are shown in Figure 2. As expected, the largest fraction (20%) consists of human *ether-à-go-go related gene* (hERG): potassium channel inhibitors known for their association with QT interval prolongation.

The majority of drugs with DICT concern could be classified as interacting with approximately 20–25 categories of targets involved in the regulation of cardiac and hemodynamic function. These include adreno, angiotensin II type 1 (AT_1_), dopamine, gamma-aminobutyric acid (GABA), histamine, muscarine, serotonin, opioid, and sex hormone (5α-reductase, androgen, aromatase, estrogen, gonadotropin-releasing hormone [GnRH], progesterone) receptors; calcium (Ca), hERG, and sodium (Na) channels; angiotensin-converting enzyme (ACE); acetylcholinesterase; cyclooxygenase (COX-1 and COX-2); phosphodiesterase (PDE); topoisomerase; tyrosine kinases; monoamine transporters (catechol-O-methyltransferase [COMT], dopamine transporter [DAT], monoamine oxidase [MAO], norepinephrine transporter [NET]), and anticoagulants/antiplatelets (Factor Xa, GP IIb/IIIa, purinergic receptor [P2Y_12_], thrombin).

Several drugs with heterogenous mechanisms of action were grouped in broader categories due to their therapeutic use. These included antineoplastics (affecting cyclin-dependent kinases 4 and 6 [CDK4/6], histone deacetylases [HDAC], poly-ADP-ribose polymerase [PARP], the mechanistic target of rapamycin [Mtor], Bruton’s tyrosine kinase [BTK], phosphoinositide-3-kinase [PI3K], tubulin, DNA alkylating, Janus kinases [JAK], and Kirsten rat sarcoma viral oncogene homolog [KRAS] targets). Additional broad categories comprised anti-infectives (including antibiotics, antivirals, antifungals, and antiparasitic drugs), glucocorticoids/immunosuppressants (affecting calcineurin, cereblon, glucocorticoid receptor [GR], and inosine monophosphate dehydrogenase [IMPDH] targets), antidiabetics (affecting dipeptidyl peptidase-4 [DPP-4], glucagon-like peptide-1 [GLP-1], and peroxisome proliferator-activated receptor γ [PPARγ] targets), as well as statins and other lipid-lowering therapies. This higher-level classification enabled further contrasting and comparison of drugs across various targets and DICT concerns. While these drugs could, in principle, be further subdivided into more specific risk target groups, such granularity was not required to illustrate the proposed conceptual framework. Indeed, the use of this broader grouping underscored the need for more detailed mechanistic classification when the aim is to identify key targets that are most informative for cardiovascular safety screening purposes.

Drugs without a direct pharmacological target, such as indocyanine green (an optical dye) and the radio-labeled positron emission tomography (PET) tracers florbetapir and flutemetamol 18F were classified into a separate category (‘No pharmacological target’). These agents are generally associated with no or less (mild) DICT concern. Although rare cases of anaphylactic shock have been reported with indocyanines, these events appear to reflect uncommon allergenic reactions rather than direct pharmacological effects on the cardiovascular system [9].

### 2.2. Drug Concentration–Response Relationships and Target Engagement

**Figure 3:**
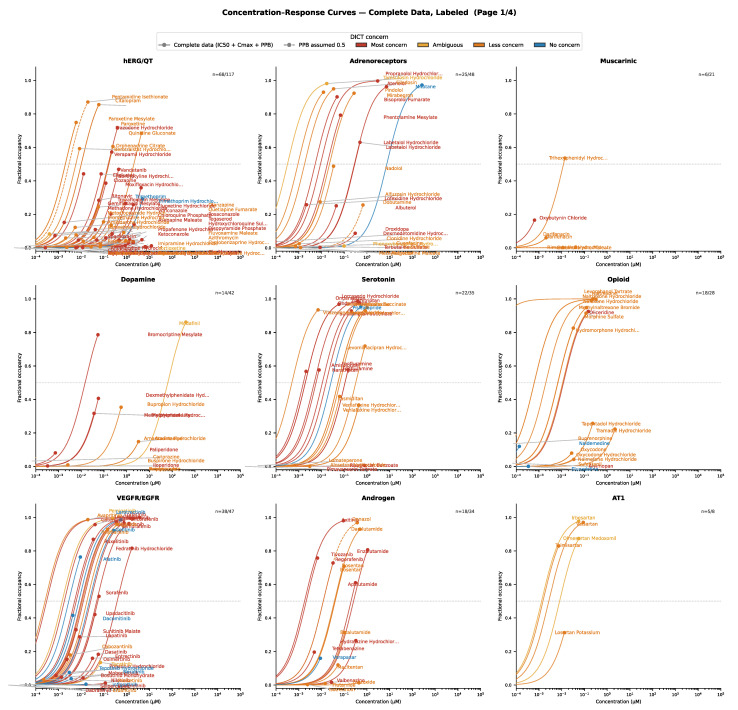
Example of drug concentration–response comparisons across various pharmacological targets frequently associated with cardiovascular effects. Circles represent the free maximum drug concentration.

**Figure 4:**
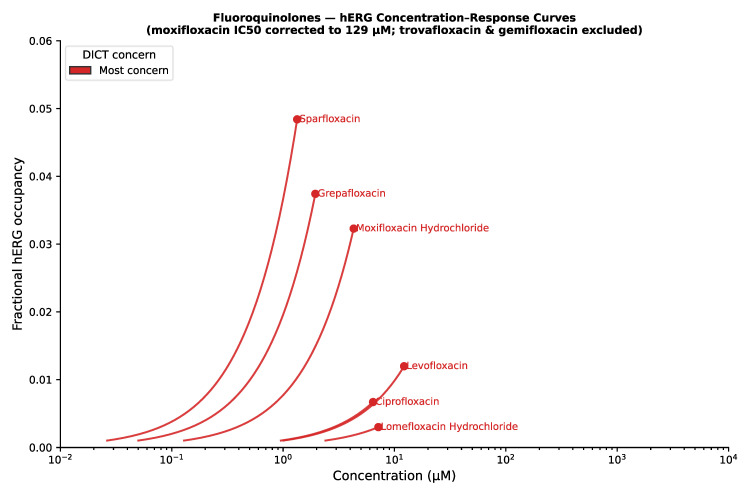
Drug concentration–response comparisons for drugs affecting the hERG channel. Circles represent the free maximum drug concentration.

**Figure 5:**
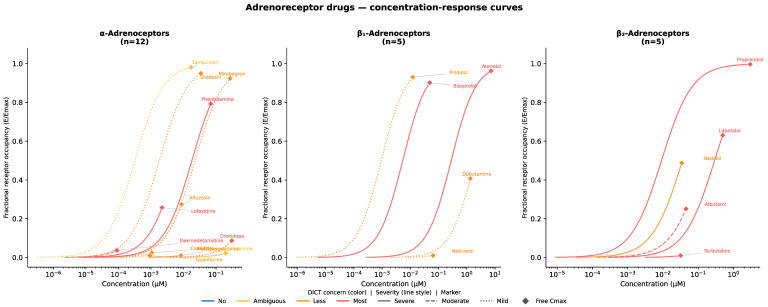
Drug concentration–response comparisons across drugs affecting adrenoreceptors. Diamonds represent the free maximum drug concentration.

**Figure 6:**
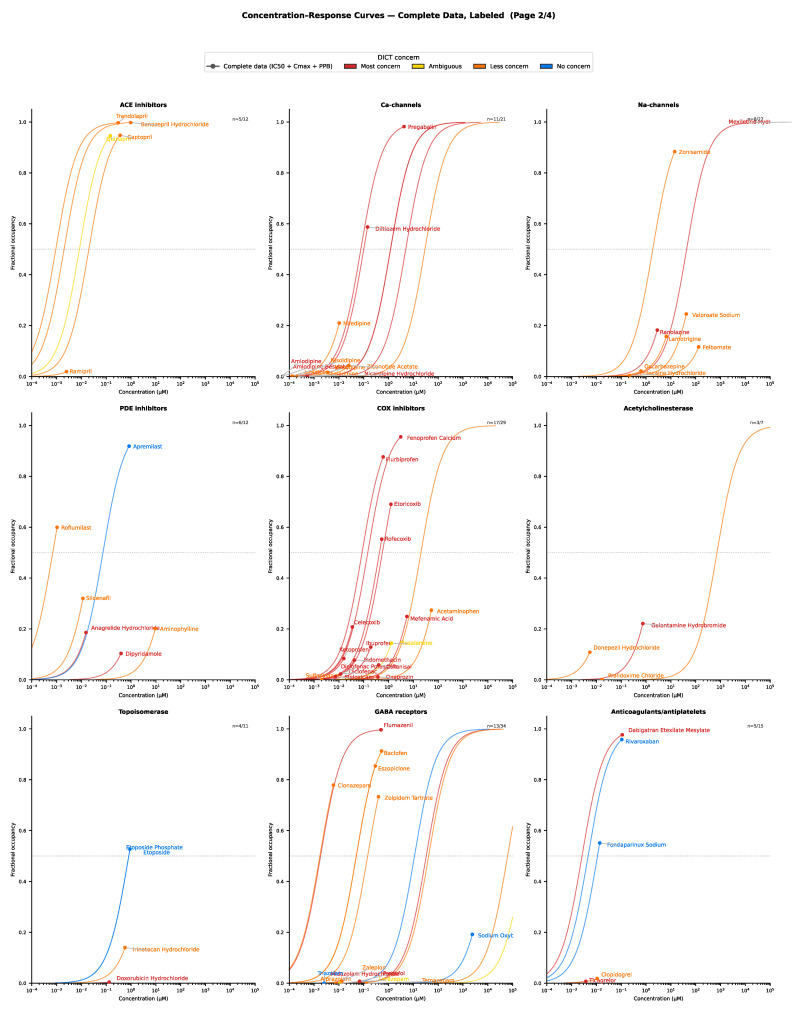
Example of drug concentration–response comparisons across other various frequently occurring drug targets associated with cardiovascular effects. Circles represent the free maximum drug concentration.

**Figure 7:**
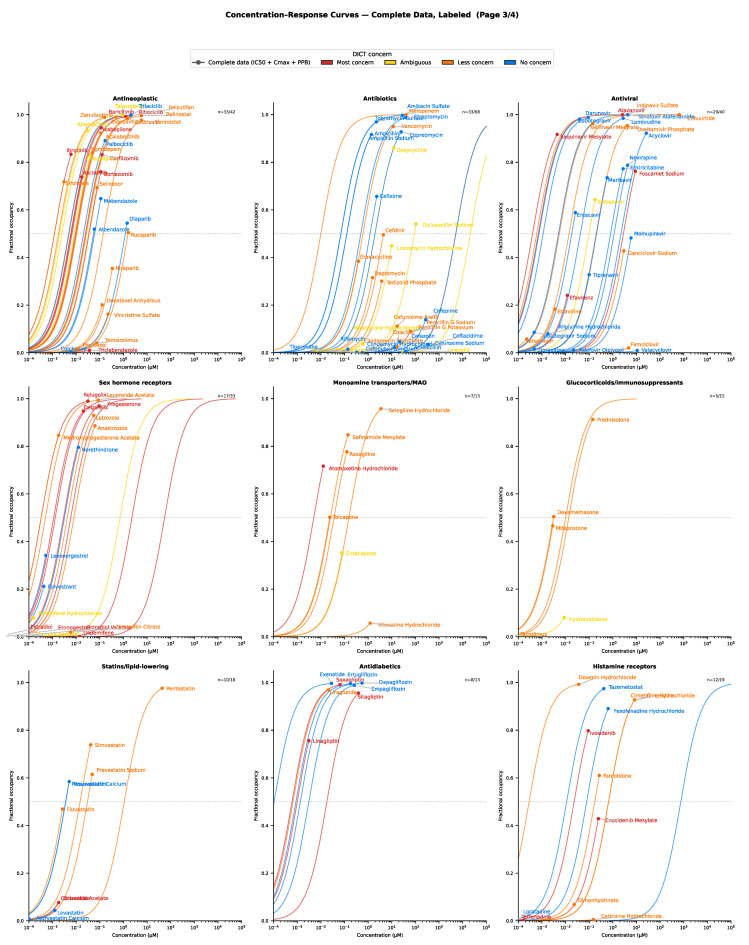
Example of drug concentration–response comparisons across classes of drugs with more diverse mechanisms of action. Circles represent the free maximum drug concentration.

**Figure 8:**
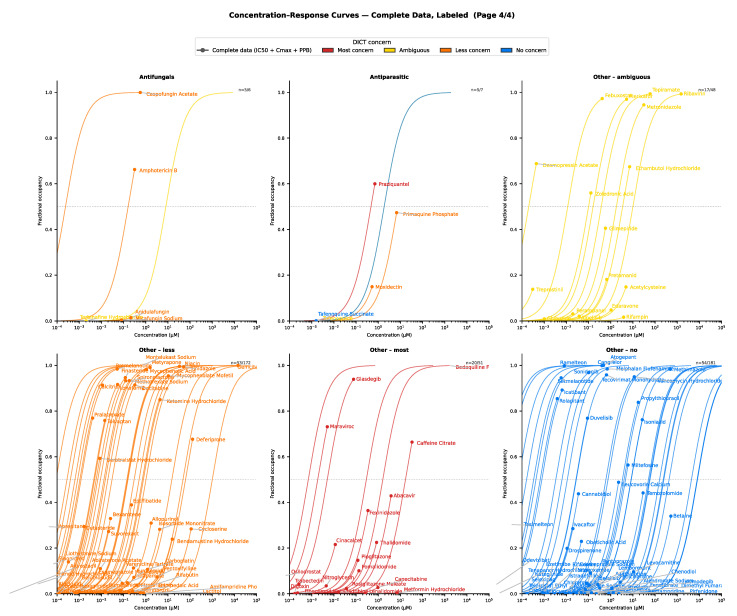
Drug concentration–response comparisons across remaining drugs in the DICTrank data affecting less frequent occurring targets. The results are stratified by DICT concern. Circles represent the free maximum drug concentration.

The retrieved pharmacological data (target affinity, plasma protein binding and C_max_) were grouped by DICT concern/severity and visualized (using maximum effect E_max_ functions), with further stratification by target class. For drugs with complete data, the concentration–response relationships for selected target classes are presented in Figure 3, Figure 4, Figure 5, Figure 6, Figure 7 and Figure 8. Targets associated with elevated cardiovascular risk were predominantly represented in panels containing a high proportion drugs classified as having safety concern and no, or only a few drugs, with no concern. In contrast, targets considered less relevant for cardiovascular risk assessment were represented in panels containing drugs of less concern or in panels showing mixed safety outcomes. Overall, these descriptive analyses revealed substantial variability in target engagement across compounds, reflecting differences in intrinsic potency and unbound clinical drug exposure. Such differences may help explain the observed variability in the DICTrank concern and severity category among drugs sharing a common pharmacological mechanism of action.

Within the hERG channel category, nearly all drugs were consistently classified as having some level of DICT concern (Figure 3). One notable exception was the antibacterial drug trimethoprim, which was classified as having no concern despite being an inhibitor of hERG. However, its predicted maximum target engagement was low, which may explain the absence of clinically relevant risk reported in the label. Among fluoroquinolones such as ciprofloxacin, moxifloxacin, and sparfloxacin, QT interval prolongation is listed as a DICTrank keyword, with concern levels ranging from less to most concern (of moderate to severe severity). Based on in vitro affinity and unbound clinical drug exposure, sparfloxacin exhibited the highest predicted target engagement, which may help explain its subsequent withdrawal from the market (Figure 4). Interestingly, the high degree of ion channel inhibition was not driven by a single factor, but rather by an untoward combination of IC_50_ value, plasma protein binding, and total C_max_. Consistent with these target engagement predictions, sparfloxacin and moxifloxacin have been reported to cause greater QT interval prolongation in clinical settings compared with ciprofloxacin (approximately 20 ms versus 0–5 ms, respectively) [10].

For several other drug classes shown in Figure 3, more pronounced discrepancies were observed. Although µ-opioid receptor agonists (like morphine and oxycodone) are well known to cause hypotension, naldemedine and eluxadoline (despite sharing the same mechanism of action) are not classified as having any DICT concern. This discrepancy may be partly explained by their lower predicted target engagement at clinically relevant drug exposures. However, it may also reflect variability in FDA labeling for these drugs, highlighting a limitation of relying on clinical outcome data alone for mechanistic characterization of adverse drug reactions.

Receptor families such as serotonin receptors comprise multiple subtypes that mediate distinct physiological effects. Drugs acting on these receptors may function as full agonists, partial agonists, or antagonists. Consequently, grouping all compounds acting within a broad receptor class can mask important subtype-specific differences. Each receptor class should therefore be evaluated with greater mechanistic granularity. For example, antimigraine triptan type 5-HT_1B_/_1D_ receptor agonists are expected to induce vasoconstriction. Consistent with this, these drugs are classified in the DICTrank database as being of most concern (causing severe reactions), with keywords including acute myocardial infarction. Although not necessarily directly causally linked, it is plausible that drug-induced vasoconstriction may increase the risk of myocardial infarction in susceptible individuals. In contrast, prucalopride, a highly selective 5-HT_4_ receptor agonist (increasing gut motility and indicated for chronic idiopathic constipation), was classified as having no DICT concern, aligning with its distinct receptor selectivity and physiological effects.

Similarly, drugs interacting with adrenoreceptors can be further stratified by receptor subtype (α or β_1_/β_2_), enabling a more granular assessment of risks related to blood pressure and heart rate (Figure 5).

Another illustrative example involves PDE inhibitors. Apremilast (a PDE4 inhibitor classified as having no concern) increases cAMP predominantly in immune cells. In contrast, PDE5 inhibitors increase cGMP in vascular smooth muscles, leading to vasodilation and distinct cardiovascular effects. For COX inhibitors, whose mechanism of action can promote vasoconstriction through reduced prostacyclin production, most drugs appear to be associated with some degree of DICT concern (Figure 6). However, the broader antiplatelets/anticoagulants category encompasses several distinct targets that warrant further mechanistic classification. For example, although both ticagrelor and cangrelor inhibit the P2Y_12_ receptor to a similar extent, only ticagrelor is classified as having DICT concern. This difference may reflect the clinical context of use where ticagrelor is administered chronically, whereas cangrelor is used for short-term treatment and exhibits rapid, reversible target binding. Similarly, dabigatran (a direct thrombin inhibitor) and rivaroxaban (a factor Xa inhibitor) both modulate the coagulation cascade to the same extent. Both should, in principle, be expected to reduce the risk of thromboembolic events. Nevertheless, only dabigatran is classified as having DICT concern. This discrepancy may reflect differences in labeling rather than true mechanistic divergence. The reported keywords for dabigatran such as ‘heart damage’ and ‘myocardial infarction’ do not appear to be directly linked to its anticoagulant mechanism of action.

Antibiotics and antivirals (with the notable exception of atazanavir, which binds to L-type calcium channels and is associated with atrioventricular [AV] block and PR interval prolongation), as well as antifungal and antiparasitic agents (those that do not interact with hERG channels [as in the case of fexinidazole] or other established risk targets), were predominantly classified as having no or less concern, typically of less severity (Figure 7). For drugs acting on sex hormone receptors, some classified as having no DICT concern (i.e., potential false positives) may benefit from further refinement at the receptor subtype level. For example, the estrogen antagonist fulvestrant, used in the treatment of breast cancer, is classified as having no concern. Its cardiovascular effects differ fundamentally from estrogen receptor agonists, which are classified as being of concern due to their associated risk of blood clotting.

Among antidiabetic drugs, DPP4 inhibitors (e.g., sitagliptin and saxagliptin) are classified as having most concern, largely due to their association with an increased risk of heart failure [11]. In contrast, sodium-glucose co-transporter-2 (SGLT2) inhibitors (e.g., dapagliflozin and empagliflozin) are classified as having no DICT concern. This distinction illustrates that broad therapeutic or disease-based classification are insufficient for safety screening. Instead, safety assessment must focus on the specific mechanism of action and exact molecular targets linked to risk. Antineoplastic agents represent a drug class with particularly high potential for further mechanistic stratification, as they encompass a wide diversity of modes of action and molecular targets with markedly different risk profiles.

**Figure A1:**
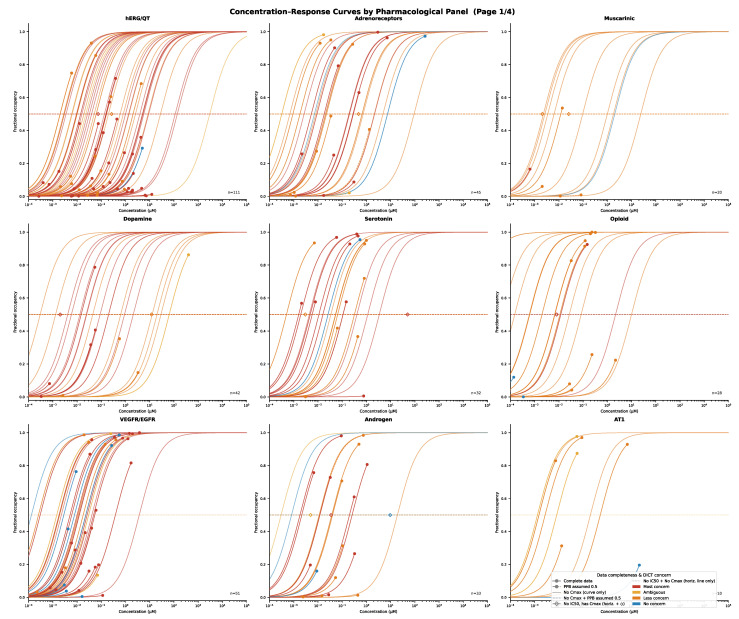

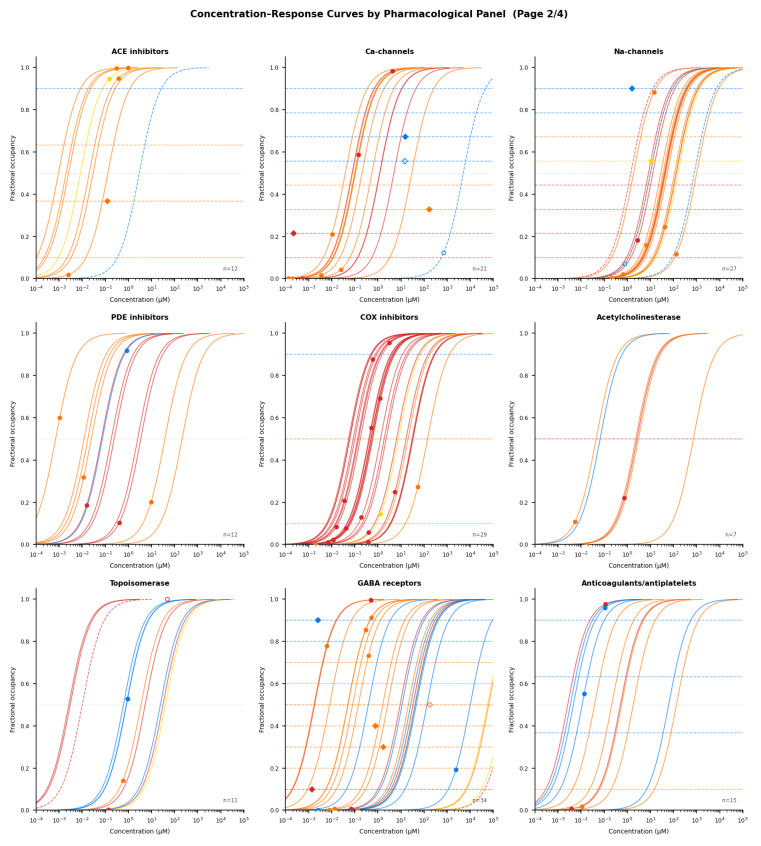

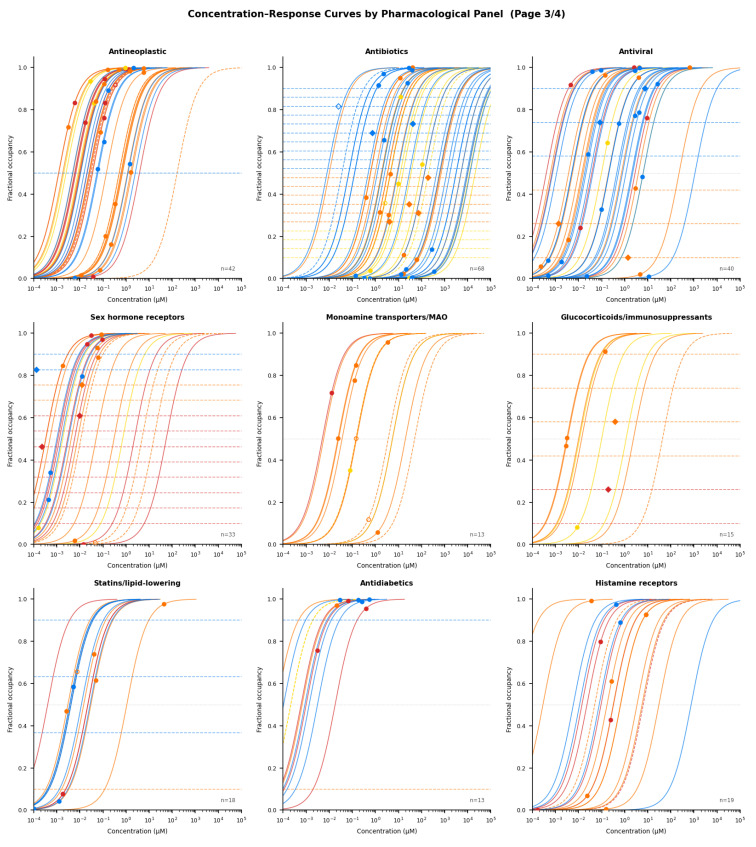

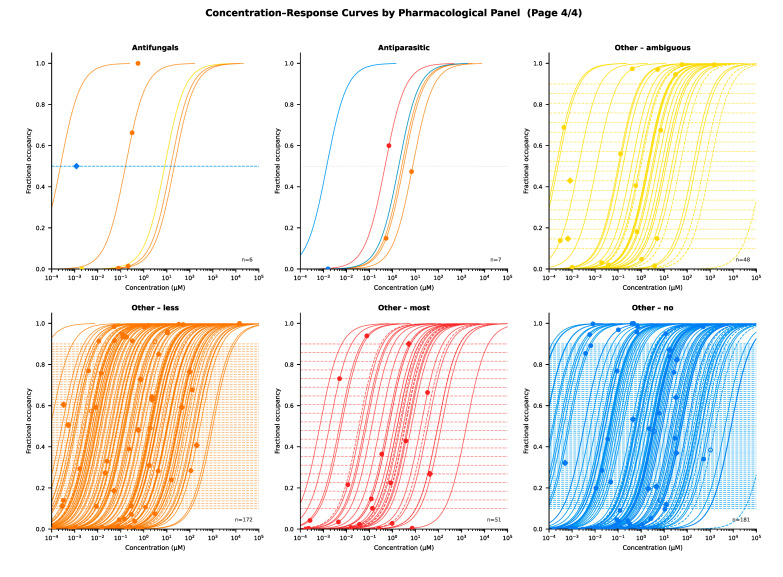
Drug concentration–response comparisons for drugs with missing parameters. Symbols represent the free maximum drug concentration (C_max_). Missing plasma protein binding was set at 0.5. Missing C_max_ values were omitted, and just the concentration–response curve was plotted. Drugs with missing affinities were plotted as horizontal lines at various *y*-axis values. Drugs with missing data should not be used for benchmarking without sensitivity analyses on the missing parameters, but were here included to further validate the selection of identified targets.

Overall, similar patterns were observed for drugs with missing pharmacological data (n = 686) as for those included in the primary analysis (Figure A1, Appendix A.1), supporting the robustness of the findings. The distribution of drugs that could not be assigned to any of the identified cardiovascular risk target classes is shown in Figure 8. Notably, only 51 of the 330 drugs of most concern were associated with alternative mechanisms of action beyond the primary targets identified in this analysis. Among these 51 high-concern drugs with other mechanisms of action, eight were contrasting agents and 19 belonged to miscellaneous categories represented by a single drug each. Although these mechanisms occur less frequently, several are well recognized for their cardiovascular effects. For example, digoxin inhibits the N^+^/K^+^-ATPase target, and caffeine interacts with adenosine receptors. In contrast, among drugs classified as having no DICT concern (n = 321), the majority were not associated with any of the major cardiovascular risk targets identified in this analysis.

### 2.3. Results Summary

Overall, 84% (276/327) of drugs classified as having the most concern, 66% (335/507) of those with less concern, and 54% (57/105) of drugs with ambiguous DICTrank toxicities could potentially be associated with one of the selected key risk target categories (excluding the ‘Other’ category). In contrast, among drugs classified as having no concern, 56% (181/321) were not assigned to any of these categories. When broader therapeutic classes (e.g., antibacterials, antivirals, antifungals, antiparasitic, antidiabetics, antihistamines and statins) were excluded, the proportion of drugs potentially linked to the risk targets only decreased modestly to 80% (260/327) for the highest concern group, 54% (275/507) for the less concern group, and 35% (37/105) for the ambiguous group. Under these more stringent criteria, only 22% (72/328) of drugs classified as having no DICT concern were potentially associated with the key risk targets. Finally, when antineoplastic, GABA-acting drugs, VEGFR/EGFR inhibitors, and anticoagulants were also excluded, only 12% of drugs classified as having no concern remained within the key cardiovascular risk target panels.

The classification could likely be further improved by examining drug classes and target types on a more detailed mechanistic level, as illustrated above for the adrenergic and serotonin receptor subtypes. More importantly, these findings demonstrate the existence of potential specific high-risk targets that may be considered for routine monitoring of new drug candidates based on in vitro affinity data.

**Figure 9:**
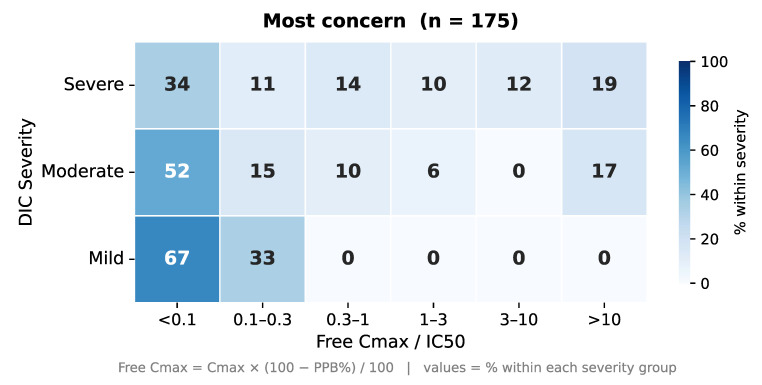
Frequency of DICT severity versus free drug concentration relative to target affinity for the most DICT concern drugs.

**Figure A2:**
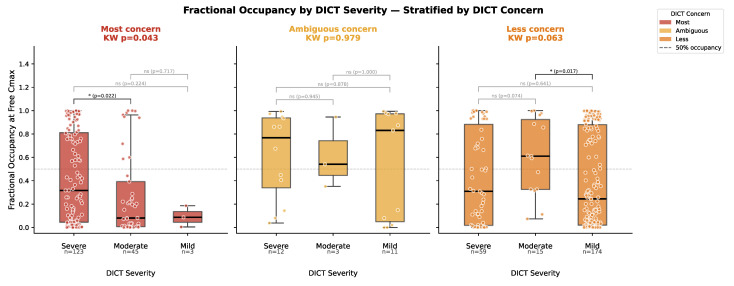
Box-and-whisker plots of target engagement versus severity of the adverse event, stratified by DICTrank concern. ns: not statistically significant. *: statistically significant. KW: Kruskal-Wallis test.

Among drugs classified as having the highest DICT concern, we observed a trend toward higher predicted target engagement for drugs associated with severe events compared with those associated with moderate events (Figure A2, Appendix A.1). This pattern was less apparent in the less and ambiguous concern categories. Ideally, each target class should be evaluated with greater mechanistic granularity and supported by more comprehensive datasets to enable robust comparisons. Nevertheless, novel drug candidates exhibiting high clinical exposure relative to their potency, particularly in combination with low plasma protein binding (e.g., free C_max_ exceeds IC_50_), should be scrutinized with particular care. Ultimately, the balance for selectivity between the therapeutic target and targets mediating adverse effects will determine the overall benefit-risk profile. Consistent with this principle, among drugs classified as having the highest DICT concern and with complete data available, drug exposure relative to target affinity was, on average, lower for drugs associated with mild events compared with those associated with severe events (Figure 9).

## 3. Discussion

We have descriptively demonstrated that in vitro drug affinity to specific cardiovascular risk targets, when adjusted for human plasma protein binding and clinical drug exposure, may potentially in many cases be associated with the safety events reported in the DICTrank database. While the underlying concept is not novel, to the best of our knowledge this is the first study to systematically apply it to a large and diverse dataset of drugs exhibiting adverse cardiovascular effects beyond QT interval prolongation. In addition, we show how publicly available pharmacological data can be integrated with clinical outcome data across multiple databases using AI-assisted literature mining. Although the framework presented here addresses only selected components of a comprehensive drug discovery and development safety program, we believe it provides a strong rationale for increased and more structured use of in vitro data to better inform subsequent animal studies and clinical trials. Overall, the proposed concept is well aligned with, and inspired by, the mechanistic framework for cardiovascular safety screening advocated by Berridge and colleagues [12].

Drugs classified as having the highest DICT concern were frequently associated with interactions with well-characterized receptors, ion channels, enzymes and transporters involved in cardiovascular on- and off-target effects. For example, approximately 20% of these drugs inhibit the hERG potassium channel, which plays a pivotal role in cardiac repolarization and whose inhibition can result in QT interval prolongation. Likewise, many other drugs associated with DICT concern interact with targets such as tyrosine kinase, dopamine, or adrenergic receptors, which are key mediators of blood pressure regulation and other cardiovascular functions.

Using an AI-assisted classification approach, we identified 18 pharmacological target classes that were frequently associated with an increased risk of adverse cardiovascular drug reactions reported in the DICTrank database. In order of decreasing frequency these include: hERG channels; adrenergic, dopamine, serotonin, androgen, sex hormone, and opioid receptors; COX, Na and Ca channels; muscarinic and glucocorticoid receptors; PDE; topoisomerase; ACE; AT_1_ receptor; monoamine transporters, and acetylcholinesterase. We also highlighted additional, less frequently represented but mechanistically relevant targets, such as the adenosine receptor and Na^+^/K^+^-ATPase. Altogether, 80% of drugs classified as having the highest DICT concern interacted with one or more of these 18 key targets. Only 12% of drugs classified as having no DICT concern were associated with these targets. Many of the identified targets are well known mediators of adverse cardiovascular effects and could therefore be routinely considered during early-stage screening of new drug candidates [13,14]. Beyond the well-established association between hERG channel inhibition and QT interval prolongation [13], several other documented drug effects have been linked to the identified targets. Endogenous catecholamines such as noradrenaline, adrenaline, and dopamine are commonly used in intensive care units to treat shock and other acute conditions due to their pronounced cardiovascular effects. However, prolonged infusion of high doses of adrenaline or noradrenaline is not recommended, as it may cause direct cardiotoxicity resulting in cardiomyocyte apoptosis or necrosis. Similarly, the nonselective β-adrenoceptor agonist isoprenaline is widely used in experimental settings to induce tachycardia; for example, when studying the effects of β-blocking drugs as well as to model a pathological state resembling acute myocardial infarction. Moreover, hormonal contraception and replacement therapies have been associated with increased risks of elevated blood pressure, myocardial infarction, and thromboembolic events. Ca channel blockers represent one of the most common causes of severe intoxication and mortality among therapeutically used cardiovascular drugs. In cases of overdose, they may induce bradyarrhythmia, systemic hypotension, and cardiovascular collapse [14].

False-negative predictions based on in vitro data may, in part, be attributable to drugs that exert toxicity through long-term chronic use, leading to gradual structural cell damage or mitochondrial dysfunction, rather than acute, target-mediated, effects. This consideration may also apply to some apparent true-positive cases, in which the identified target serves mainly as a surrogate marker for parallel or downstream adverse pharmacological processes rather than being the direct cause of toxicity. Additional contributors may include downstream off-target effects, immune-mediated mechanisms, or idiosyncratic drug reactions, all of which require further investigation. For drugs associated with rare adverse events, individual patient susceptibility and variability in drug exposure (e.g., due to genetic polymorphism in drug metabolizing CYP450 enzymes) may also play an important role in determining cardiovascular risk.

Limitations of this work include reliance on currently available public data, potential biases in adverse drug reaction classification, limited representation of clinical between-subject variability, and the absence of prospective validation. Reliance on clinical binary outcome data for mechanistic characterization of adverse drug reactions represents a general limitation. Ideally, longitudinal repeated-measures endpoint data at the individual level should be included to better characterize the relationship between drug concentrations and response over time, including quantification of variability. Accurate prediction of drug exposure and the cardiovascular response at the upper end of the population distribution is critical for making reliable safety judgements.

It should be noted that the DICTrank database contains some inconsistencies, as it relies on drug-specific labels which are naturally not fully harmonized for comparative benchmarking. For some drugs within the same class, either primary or secondary pharmacological effects may be reported inconsistently across labels. For example, sildenafil and sildenafil citrate (representing the same active drug inhibiting the PDE5 enzyme, causing cGMP increases and vasodilation) are associated with different adverse event keywords (hypotension versus heart failure and tachycardia) and assigned different severity classifications (mild versus severe). Similarly, prazosin and other adrenergic α-1 receptor antagonists are in some cases reported to cause vasodilation, whereas in others increased heart rate is emphasized. This likely reflects the underlying physiological sequence, in which an initial reduction in blood pressure may trigger compensatory tachycardia. Such variability in labeling underscores an inherent limitation of relying solely on clinical adverse event annotations for mechanistic benchmarking. Likewise, for drugs associated with multiple adverse event keywords, we prioritized QT interval prolongation over other reported endpoints. This prioritization may have led to a relative overestimation of hERG channel inhibition contribution among the identified risk targets.

Another limitation relates to the potential contribution of active metabolites to cardiovascular adverse drug reactions. For example, in the case of the antiparasitic drug fexinidazole, the risk of QT interval prolongation has been linked primarily to the plasma concentration of its active metabolite, fexinidazole sulfone (M2), rather than to the parent compound. Consequently, target affinities, plasma protein binding, and drug exposure should, in some cases, also be evaluated for relevant metabolites in order to accurately assess cardiovascular risk.

It should be emphasized that the objective of the present work was not to provide a comprehensive mechanistic explanation of all forms of cardiovascular toxicity, but rather to demonstrate how pharmacological classification approaches can support a more precise understanding, mapping and quantification of cardiovascular risk. Advances in AI and machine learning, when combined with established principles of classical pharmacology, have the potential to significantly transform drug discovery and development. Tools such as those illustrated here can facilitate and accelerate the benchmarking of new drug candidates against competitors and marketed drugs, particularly for broader project teams that may lack specialized data-programming expertise. In this context, AI-assisted literature mining proved to be an efficient means of compiling and integrating pharmacological and clinical information from diverse sources. Some of the missing drug-specific data not retrieved in this work can possibly be recovered through additional targeted search iterations and manual curation, further strengthening the utility of the proposed framework.

In addition, the retrieved target affinity and pharmacokinetic data may be subject to measurement errors and selection bias. Reported target affinity values can vary substantially depending on the experimental assay used and should therefore be interpreted with appropriate caution. Given that the primary objective of the present work was to establish a conceptual framework rather than to provide an exhaustive quantitative characterization, the available data were considered sufficient to illustrate the underlying principles. However, if this framework is applied for benchmarking new drug candidates against known compounds, further cross-validation and systematic comparisons across multiple data sources would be advisable to enhance robustness and confidence in the results.

When applied prospectively during the early stages of discovery and development stages, this framework enables an initial assessment of cardiovascular risk using in vitro data alone. By identifying compounds with unfavorable risk profiles early, the approach has the potential to reduce the need for subsequent animal studies by preventing the progression of high-risk candidates. For compounds that do advance to the next development phase, animal studies can be refined through improved study design and more informed dose selection guided by in vitro data [15]. The mechanistic nature of the framework supports more nuanced assessments than binary (yes/no) safety classification. It enables quantitative comparison between molecules and can guide prioritization during lead selection and optimization. Expected target engagement can be benchmarked against marketed drugs or competitors with known cardiovascular safety liabilities, thereby supporting informed go/no-go decision and investment prioritization.

Following prospective validation of these findings, routine screening of new drug candidates for affinity to the pharmacological targets identified here as potentially being associated with cardiovascular risk may be considered. For reproducibility, the relevance of each identified key risk target could be further evaluated by augmenting the corresponding target categories with additional drugs. When the expected therapeutic exposure (especially in the upper end of the population), in combination with low plasma protein binding, exceeds the estimated target potency, there may be substantial risk of cardiovascular effects. For robust and transparent decision-making, more detailed target-specific frameworks should be developed. These frameworks should define acceptable safety thresholds and decision criteria, while recognizing that such thresholds will ultimately depend on the therapeutic indication and level of unmet medical need. A recently proposed framework, integrating drug structure, tissue exposure, selectivity, and activity relationships, classifying drugs in four categories based on potency and exposure [16], could be applied in conjunction with the approach described here.

Computational medicinal chemistry models have the potential to elucidate which molecular structural features drive high affinity toward targets associated with cardiovascular risks. By identifying specific high-risk targets, our classification may facilitate the discovery of target-specific pharmacophores of risk. There is a clear need for additional user-friendly in silico methods capable of predicting how candidate molecules might be modified to mitigate cardiac toxicity while preserving their desired therapeutic effects.

Future internal validation could include expanding the dataset to include target affinities to all the identified key targets for each drug, rather than limiting the analysis to the most plausible mechanism underlying the reported event. This could help to better quantify the risk of false-positive associations. In parallel, the proposed classification system should be evaluated using alternative external data sources. Planned future work includes validation of the framework using data from the FDA’s adverse event reporting system. Because the DICTrank data is inherently biased toward drugs that have reached the market, additional insights may be gained from historical pharmaceutical industry data on compounds that failed during development. In a subsequent phase, prospective evaluation of the framework would be valuable to assess false-negative rates and quantify its impact on development timelines and cost. More detailed characterization of longitudinal adverse event data, combined with model-based analyses aimed at identifying critical target engagement thresholds for specific risk targets, could further improve the predictive performance and practical utility of the framework.

In the present work we focused on primary pharmacology and target engagement, such as receptor occupancy or enzyme inhibition. Quantifying the relationship between target engagement and the subsequent time course of downstream pharmacological responses could enable more precise prediction of safety risks and their translation into clinically relevant endpoints. From a systems biology and pharmacology perspective, a key determinant of drug-induced cardiotoxicity is the number, type, and magnitude of perturbations a compound induces across cardiac signaling networks. Drugs that disrupt multiple pathways or central network nodes are more likely to produce system-level adverse effects, even when single-target potency or exposure metrics appear unremarkable. Therefore, complementing the pharmacological classification approaches presented here with assessment of network-level perturbations may substantially improve the prediction of cardiovascular toxicity. With respect to plasma protein binding, it should be noted that for intracellular targets (e.g., topoisomerases, many kinases), unbound plasma concentration may not accurately reflect unbound concentration at the target site of action. Processes such as active transport, lysosomal trapping, or protein binding in tissues can result in discrepancies between systemic and intracellular drug exposure. This consideration is particularly relevant for drugs with a high volume of distribution and may necessitate more advanced frameworks for evaluating cardiovascular risk.

Finally, the methodology presented here is broadly applicable to other safety assessments (e.g., hepato-, nephro- and CNS toxicities), and can also be used for therapeutic benchmarking when there are competing drugs interacting with the same pharmacological target. Many general drug targets (n = 44) are already routinely screened within the pharmaceutical industry [17]. We encourage pharmaceutical and biotechnology companies to further expand these practices by illustrating how in vitro data (beyond hERG inhibition) can be systematically leveraged for safety assessments. The intended context of use is early discovery and preclinical optimization, rather than regulatory decision-making. Looking ahead, regulatory guidelines on the specific targets for which in vitro data should be generated could be further expanded, analogous to the evolution of the comprehensive in vitro proarrhythmia assay (CiPA) initiative [18]. A recent motivating example in this direction is the use of Ca channel in vitro data to predict changes in human cardiac contractility. In that work, the authors also outline a pathway for its integration as part of a preclinical drug safety assessment strategy, which could be extended to other targets described in the present work. Furthermore, the authors also emphasize the need for additional data to establish sets of reference compounds and highlight the potential value of combining in vitro data with more mechanistic tissue and organ scale modeling frameworks [19]. The growing integration of novel approach methodologies has the potential not only to reduce reliance on animal studies, but also to decrease development cost and timelines, ultimately enabling the delivery of more effective and safer medicines to patients. Conducting comprehensive in vitro-based safety assessments at an early stage, prior to initiating animal studies or clinical trials, can substantially improve the design and efficiency of new drug development programs.

## 4. Materials and Methods

### 4.1. Data

The DICTrank dataset was downloaded from the FDA website (https://www.fda.gov/science-research/bioinformatics-tools/drug-induced-cardiotoxicity-rank-dictrank-dataset, accessed on 10 May 2026). The dataset covers diverse therapeutic categories and includes drugs with a single active ingredient approved for human use by the FDA. A total of 1318 drugs (1291 FDA-approved drugs plus 27 withdrawn drugs) were classified into four groups depending on their DICT potential: most, less, no and ambiguous DICT concern. These classifications were derived based on descriptions of cardiotoxicity in the various drug label sections. In addition to classifications of overall level of concern and severity (mild, moderate, severe), the dataset includes information on the type of cardiotoxicity (arrhythmia, heart damage or mixed) as well as adverse event-related keywords (e.g., hypertension, tachycardia, or QT prolongation). The drugs were distributed across the DICT concern categories as follows: most concern (n = 341), less concern (n = 528), ambiguous concern (n = 106) and no concern (n = 343). The dataset has been described in more detail by Qu et al. [2]. In the original dataset, several drugs appeared multiple times under different trade names or salt forms. In our analysis, we therefore chose to consolidate the data to include only unique generic drug names. In total, 1267 unique generic drug names from the DICTrank database were included in this analysis.

### 4.2. AI-Assisted Literature Research

We enriched the DICTrank dataset by assigning a plausible pharmacological target (such as a receptor, ion channel, enzyme or transporter) whose modulation could reasonably explain the association with the adverse cardiovascular event based on an extensive review of the published literature. For each drug, we additionally retrieved corresponding target affinity, human plasma protein binding (fraction unbound), and clinical exposure metrics (here defined as maximum plasma concentration [C_max_]) based on literature data.

The AI-assisted literature search was automatically conducted using Biomni v0.0.8 (Phylo, https://www.phylo.bio) between February and April 2026, drawing on publicly available sources such as ChEMBL, PubChem, Binding DB, DrugBank, drug product labels, etc., as determined appropriate and accessible by the tool [20]. All analyses were performed in Python 3.11 using pandas, numpy, matplotlib, and requests. The search criteria were to ‘classify the drugs (using the generic names) in the DICTrank dataset based on what on- or off-target receptor, ion channel, enzyme or transporter which the drug interacts with and whose plausible modulation may be associated with the reported cardiac or hemodynamic response’. Classification was applied using a three-tier hierarchical approach. For each drug individually, the primary pharmacological target was identified from the biomedical literature and/or pharmacology databases. Target type, on- or off-target cardiac effect classification, and a mechanistic description connecting the target to the observed cardiovascular effect were added. For drugs belonging to a well-characterized pharmacological class with stereotyped cardiovascular mechanisms, a class-level rule could be applied rather than an individual drug-by-drug lookup. For drugs with well-characterized target binding profiles, the target was pulled directly from ChEMBL. Drug affinities and exposure metric units were harmonized to nM, using molecular weight information where necessary. When multiple values were identified, the median was used. The full search prompt is summarized in Appendix A.2. Retrieved pharmacological data underwent a brief manual quality control through checking of the amended records for each individual drug. The proposed target, the pharmacological mechanism of action and the expected primary cardiac and hemodynamic effects were reviewed via human-in-the loop verification to assess plausibility. Corrections were made where needed. Each target affinity, drug exposure, and plasma protein binding value were not manually controlled and may contain inconsistencies and errors. The data should therefore be interpreted with appropriate caution but were considered sufficient to illustrate the underlying principles of the framework.

During manual quality control several inconsistencies were identified. For many of the drugs classified in the hERG/QT category, the reported IC_50_ values reflected affinity for the therapeutic on-targets rather than inhibition of the hERG channel relevant for the safety concern. Examples include dopamine receptor antagonism for asenapine and enhancement of calcium sensing receptor sensitivity for cinacalcet, rather than hERG inhibition. In the case of moxifloxacin, the target was incorrectly listed as DNA gyrase (the therapeutic target). This was manually corrected to hERG channel inhibition, with an IC_50_ of 129 µM [21]. Comparable misassignments may have occurred for drugs in other classes. These issues may have contributed to inaccuracies in the shape of certain exposure–response curves. However, they were considered unlikely to affect the overarching objective of illustrating the proposed conceptual framework. In addition, a scripting error initially caused several opioid drugs to be incorrectly classified and plotted within the muscarinic receptor panel. This issue was subsequently identified and updated by the Biomni tool. Furthermore, probenecid (a uric acid transporter 1 inhibitor of no DICT concern) was incorrectly classified into the AT_1_ category. Unlike true AT_1_ receptor antagonists (such as irbesartan and valsartan which are associated with hypotension), probenecid has no such mechanism and was therefore moved to the ‘Other no concern’ category.

### 4.3. Data Visualization and Dose Exposure–Response Characterization

The resulting data were sorted (by DICT concern and severity categories) and summarized according to the primary drug target classes considered most likely to drive the reported adverse effects based on the literature review. Exposure–response relationships were derived for each drug:

Fractional target engagement = (E_max_ × C)/(EC_50_ + C), (1)

where C is the drug concentration and EC_50_ (IC_50_ for antagonists and inhibitors) is the affinity to the target (the concentration where half the maximum engagement occurs) based on in vitro data. The maximum effect parameter (E_max_ for agonists and I_max_ for antagonists) was set to 1 for all drugs, arbitrarily symbolizing full target engagement. The free maximum unbound clinical drug concentration (C_max,u_) was highlighted for each drug. It was derived by combining human plasma protein binding and total clinical exposure (C_max_):

C_max,u_ = C_max_ × (1 − plasma protein binding). (2)

In this exploratory, hypothesis-generating, descriptive analysis, the exposure–response curves were visualized and stratified into panels according to shared or similar target classes (as identified by the literature review), enabling direct comparison of relative target potency and clinical drug exposure across compounds. The aim was to examine approximately 20–25 common target classes considered of potential relevance for cardiovascular safety screening purposes and to illustrate the proposed conceptual framework. The selected target classes should not be considered exhaustive but represent those most frequently associated with reported cardiovascular risk in the analyzed dataset. Several drugs, with heterogenous mechanisms of action, were grouped in broader categories due to their therapeutic use. These explorations included antineoplastics, anti-infectives, glucocorticoids/immunosuppressants, antidiabetics as well as statins and other lipid-lowering therapies. This higher-level classification enabled further contrasting and comparison of drugs across various targets. For some target classes, such as adreno and serotonin receptors, we also explored further stratification by receptor subtype to enable a more granular assessment of risks.

Remaining drugs, acting on less common or less clearly relevant targets and other therapeutic classes, were grouped separately and displayed in four panels according to their DICT concern category. In brief, panels containing a higher number of drugs of most concern were considered more interesting for screening purposes, whereas panels dominated by drugs of no concern were considered less suitable for early safety risk assessment. Panels showing mixed outcomes (i.e., containing drugs both with and without any concern) were not deemed sufficiently specific for direct screening but were interpreted as warranting further subclassification.

Because some drugs mapped to multiple pharmacological targets and adverse event types, drugs affecting hERG (or listed with QT interval prolongation or Torsades des Pointes as keywords) were assigned a priori to the hERG panel, reflecting the well-established role of this target in cardiovascular safety. This prioritization was applied even when additional potential mechanisms were present. For example, asenapine (a dopamine D_2_ receptor antagonist, also affecting the α_1_ adrenergic receptor and hERG channels) may induce both hypotension and QT prolongation. Although it could reasonably be classified under multiple categories, it was assigned to the hERG panel to ensure clarity, consistency and focus of the analysis. All graphical visualizations were generated using Biomni.

### 4.4. Association Between Exposure–Response and DICT Concern

The exposure–response relationships were subsequently compared with the cardiac and hemodynamic outcomes described in the FDA labels. Apparent associations between the extent of predicted target engagement and DICT concern category (as well as the severity of the event) were assessed descriptively.

Primary analyses were first performed using only drugs for which complete pharmacological and exposure data were available. Eventually, all drugs with missing literature data were included in a sensitivity analysis. For drugs lacking plasma protein binding, fraction unbound was set at 0.5. Missing C_max_ data was omitted from the plot, and just the full concentration–response curve was plotted. Drugs with missing target affinity were visualized as horizontal lines at various *y*-axis values. The purpose of this sensitivity analysis was to evaluate whether the overall patterns and target class assignments observed in the primary analysis remained consistent when including more data, rather than to enable detailed quantitative comparison between drugs with complete and incomplete data.

## 5. Conclusions

We used the DICTrank database to explore potential drug targets and pharmacological mechanisms potentially underlying a broad range of reported adverse cardiovascular events. Through this exploratory, hypothesis-generating, descriptive analysis, we identified 18 classes of risk targets potentially associated with elevated cardiovascular risk that may be considered in early safety assessment of new drug candidates after prospective validation.

Machine learning-based methods hold great promise for distinguishing molecular properties associated with adverse cardiovascular drug reactions. However, for such methods to be useful in translational and clinical contexts, they must be explainable and firmly grounded in mechanistic understanding and established pharmacology principles. Accordingly, for any drug candidate, affinity to key receptors, ion channels, enzymes and transporters may be evaluated in conjunction with therapeutic exposure metrics (including the impact of between-subject variability) and human plasma protein binding.

## Data Availability

The original contributions presented in this study are included in the article. Further inquiries can be directed to the corresponding author.

## Abbreviations

The following abbreviations are used in this manuscript:

5-HT	5-hydroxy tryptamine (serotonin)
AI	Artificial intelligence
ACE	Angiotensin-converting enzyme
ADMET	Absorption, distribution, metabolism, excretion, toxicity
AT_1_	Angiotensin II type-1
AV	Atrioventricular
BTK	Bruton’s tyrosine kinase
Ca	Calcium
CDK4/6	Cyclin-dependent kinases 4 and 6
CiPA	Comprehensive in vitro proarrhythmia assay
C_max_	Maximum drug concentration
COMT	Catechol-O-methyltransferase
COX	Cyclo-oxygenase
CYP	Cytochrome P450
DAT	Dopamine transporter
DICT	Drug-induced cardiac toxicity
DICTrank	DICT rank database
DPP-4	Dipeptidyl peptidase enzyme
EC_50_/IC_50_	Drug concentration where half of the target engagement is achieved
EGFR	Epidermal growth factor
GABA	Gamma-aminobutyric acid, neurotransmitter
GLP-1	Glucagon like peptide-1
GnRH	Gonadotropin releasing hormone
GR	Glucocorticoid receptor
HCN	Hyperpolarization-activated cyclic nucleotide-gated, ion channel
HDAC	Histone deacetylases
hERG	Human *ether-à-go-go related gene*, coding for the voltage-gated potassium ion channel
IMPDH	Inosine monophosphate dehydrogenase
JAK	Janus kinases
KRAS	Kirsten rat sarcoma viral oncogene homolog
MAO	Monoamine oxidase
Mtor	The mechanistic target of rapamycin
Na	Sodium
NET	Norepinephrine transporter
K	Potassium
PARP	Poly-ADP-ribose polymerase
PET	Positron emission tomography
PPARs	Peroxisome proliferator–activated receptors
P2Y_12_	Purinergic receptor
PDE	Phosphodiesterase enzyme
PI3K	Phosphoinositide-3-kinase
RAAS	Renin–angiotensin–aldosterone system
SGLT2	Sodium-glucose co-transporter
VEGF	Vascular endothelial growth factor

## Appendix A

### Appendix A.1

Panels including all drugs, also encompassing those with missing C_max_, plasma protein binding, or target affinity are shown in Figure A1. In total, 1267 drugs were included. Complete data for all variables were available for 581 drugs. Among the remaining compounds, 251 drugs lacked C_max_ values, 79 drugs lacked both C_max_ and plasma protein binding values, and an additional 24 lacked plasma protein binding. Only 73 drugs had no EC_50_/IC_50_ values. A total of 259 drugs lacked both EC_50_/IC_50_ and C_max_ data. Box-and-whisker plots illustrating target engagement stratified by DICTrank severity for drugs with complete datasets are presented in Figure A2.

### Appendix A.2

#### Construction of the Extended DICTrank Pharmacological Dataset

Overview

Using Biomni, the DICTrank dataset of FDA-labeled drugs with drug-induced cardiotoxicity (DICT) annotations was extended with four categories of pharmacological and pharmacokinetic (PK) data: cardiac target classification, plasma protein binding (PPB), clinical maximum plasma concentration (C_max_), and in vitro target affinity (IC_50_/EC_50_). All data retrieval, parsing, and integration were performed programmatically in Python. The final dataset comprises 1318 entries corresponding to 1267 unique drugs.

Cardiac Target Classification

Each drug was annotated with its primary cardiac molecular target (receptor, ion channel, or enzyme), target type, on- or off-target cardiac effect classification, and a mechanistic description. Classification was applied using a three-tier hierarchical approach.

Tier 1—Expert-Curated Pharmacology Lookup

The primary classification source was a manually curated reference dictionary covering all drugs in the dataset. Each entry specified the molecular target using standard nomenclature (e.g., “hERG (KCNH2) K^+^ channel”, “β1 adrenergic receptor (ADRB1)”), target type (receptor, ion channel, or enzyme), whether the cardiac effect was on- or off-target, and a brief mechanistic description. All dictionary keys were normalized to uppercase for case-insensitive matching.

Tier 2—ChEMBL Mechanism-of-Action Database

For drugs not covered by the curated dictionary, the ChEMBL REST API (v33) was queried for mechanism-of-action records. Retrieved target names were matched against a library of regular expression patterns covering cardiac-relevant molecular targets (ion channels, adrenergic receptors, muscarinic receptors, angiotensin-converting enzyme, HMG-CoA reductase, and others). Matched records were used to populate the target annotation fields.

Tier 3—Drug-Class Name Pattern Matching

For remaining unclassified drugs, target class was inferred from the International Nonproprietary Name (INN) using established suffix and stem conventions (e.g., “-olol” → β-adrenergic receptor blocker; “-pril” → angiotensin-converting enzyme inhibitor; “-statin” → HMG-CoA reductase inhibitor).

All 1318 entries received a classification. Five columns were added to the dataset: Cardiac Target (Receptor/Channel/Enzyme), Target Type, On/Off-Target Cardiac Effect, Cardiac Mechanism, and Classification Source.

For downstream visualization, the free-text cardiac target strings were mapped to 20 major pharmacological classes: hERG (IKr) K^+^ channel; voltage-gated Na^+^ channel (Nav); voltage-gated Ca^2+^ channel (Cav); β-adrenergic receptor; α-adrenergic receptor; ACE/angiotensin (renin–angiotensin–aldosterone system [RAAS]); muscarinic receptor; dopamine receptor/transporter; serotonin receptor/transporter; opioid receptor; histamine receptor; kinase (RTK/oncology); COX (cyclooxygenase); HMG-CoA reductase; GABA receptor; topoisomerase/DNA damage; Na^+^/K^+^-ATPase; phosphodiesterase (PDE); coagulation/antiplatelet; antimicrobial/antiviral. Assignment was based on first-match priority against a ranked pattern list applied to the target string.

Plasma Protein Binding

Plasma protein binding (PPB, expressed as percentage bound) was retrieved from three sources in the following priority order.

ChEMBL

The ChEMBL activity endpoint was queried for each drug using its ChEMBL compound identifier, filtering for standard_type  =  “PPB”. Values were converted to percentage units where necessary.

PubChem PUG-View API

For drugs without a ChEMBL PPB record, the PubChem PUG-View JSON endpoint was queried using the drug’s CID. The full compound record was searched recursively across all pharmacology, pharmacokinetics, ADME, and toxicokinetics sections. Percentage protein binding values were extracted using regular expressions designed to capture a range of reporting formats (e.g., “approximately 95% bound”, “protein binding: 80–90%”).

openFDA Drug Label API

For drugs still lacking a PPB value, the openFDA drug label API was queried using the generic name, brand name, and active substance name as search terms. The clinical_pharmacology and pharmacokinetics label sections were searched with the same regular expression patterns.

When multiple PPB values were retrieved for a single drug from a single source, the median was used. The source-priority hierarchy (ChEMBL  >  PubChem  >  openFDA) was applied such that a value from a higher-priority source always superseded one from a lower-priority source. Final PPB coverage: 1028 of 1267 unique drugs (81.1%).

Clinical Maximum Plasma Concentration (C_max_)

C_max_ values were retrieved from the same three sources and priority hierarchy as PPB. All retrieved values were converted to two standard units: ng/mL and μM. Unit conversion followed these rules: ng/mL (×1), μg/mL (×1000), mg/L (×1000), pg/mL (×0.001), ng/L (×0.001). For molar units (nM, μM, mM), conversion to ng/mL used the drug’s molecular weight retrieved from ChEMBL:

ng/mL =  concentration (μM) × MW (g/mol).

Conversion to μM used:

μM = ng/mL ÷ MW (g/mol).

Values reported in non-plasma units (e.g., ng/g tissue, ng/h, ng/mL/mg dose-normalized) were excluded. When multiple C_max_ values were available from a single source, the median was used. Final C_max_ coverage: 698 of 1267 unique drugs (55.1%).

In Vitro Target Affinity (IC_50_/EC_50_)

In vitro target affinity values were retrieved exclusively from the ChEMBL activity endpoint. For each drug with a ChEMBL compound identifier, all activity records with assay_type of binding (B) or functional (F) were retrieved. Primary assay types were IC_50_ and EC_50_; Ki, Kd, Kb, AC50, and Potency were used as fallbacks when no IC_50_/EC_50_ records were available. All activity values were converted to nanomolar (nM) units (nM × 1; μM × 1000; mM × 1,000,000; pM × 0.001).

Target Matching

Retrieved records were filtered to retain only those whose ChEMBL target name matched the drug’s classified cardiac target (Section 2.1). Matching was performed using a keyword scoring system (score 0–4): score 4 indicated a direct substring match between the ChEMBL target name and the cardiac target string; score 3 indicated a match to a curated keyword pattern for that target class; score 2 indicated word-level overlap. Records with a score of zero were retained only if they represented the most frequently reported target across high-confidence assay types (IC_50_, EC_50_, Ki, Kd).

Exclusion Criteria

Records were excluded if the ChEMBL target was annotated as “Unchecked” or “NON-PROTEIN TARGET”, or if the target name contained terms indicating a non-mammalian organism (e.g., Toxoplasma gondii, Trypanosoma cruzi, Plasmodium falciparum, SARS-CoV-2, rabbit, rat, mouse) or a cell line (e.g., HeLa, MDA-MB-231). AC50, Potency, and GI50 records were only accepted if the target match score was ≥2 to prevent spurious hits from phenotypic panel screens.

Aggregation

The median was taken across all retained measurements for each drug–target pair. Final affinity coverage: 938 of 1267 unique drugs (74.0%). Five columns were added: IC_50_/EC_50_ (nM), Affinity Type, Affinity Target (ChEMBL), Affinity N Measurements, and Affinity Source.

Pharmacodynamic Occupancy Curves

To contextualize in vitro affinity relative to clinical exposure, concentration–effect (E_max_) curves were generated for all drugs with both a free C_max_ and an IC_50_/EC_50_ value (n  =  325 drugs across 19 target classes). The Hill equation with Hill coefficient n  =  1 was used:

E(C) = C/(EC_50_ + C),

where E is the fractional effect (E_max_ normalized to 1), C is the drug concentration (μM), and EC_50_ is the in vitro IC_50_ or EC_50_ (converted from nM to μM).

Free C_max_ was calculated as:

C_max,free_ = C_max,total_ × (1  − PPB/100),

where C_max,total_ is the clinical total plasma C_max_ (μM) and PPB is the percentage plasma protein binding.

Curves were plotted over a concentration range of 0 to 3 × EC_50_ for each drug, providing a standardized pharmacodynamic window centered on the EC_50_. The free C_max_ was marked on each curve with a diamond symbol (◆); drugs whose free C_max_ exceeded 3 × EC_50_ (i.e., predicted fractional occupancy > 75%) were indicated with a rightward arrow at the axis boundary. The *x*-axis was clipped at the 90th percentile of 3 × EC_50_ values within each target class to prevent outliers from compressing the majority of curves.

Curves were color-coded by DIC severity level (red: severe; orange: moderate; green: mild; gray: unknown) and distinguished by line style according to DICTRANK concern category (solid: most concern; dashed: less concern; dotted: no concern; dash-dot: ambiguous). One figure was generated per major target class.

All analyses were performed in Python 3.11 using pandas, numpy, matplotlib, and requests. Data were retrieved from ChEMBL (v33), PubChem, and openFDA APIs between February and April 2026.
